# Extremely High Frequency Electromagnetic Fields Facilitate Electrical Signal Propagation by Increasing Transmembrane Potassium Efflux in an Artificial Axon Model

**DOI:** 10.1038/s41598-018-27630-8

**Published:** 2018-06-18

**Authors:** Simona D’Agostino, Chiara Della Monica, Eleonora Palizzi, Fabio Di Pietrantonio, Massimiliano Benetti, Domenico Cannatà, Marta Cavagnaro, Dariush Sardari, Pasquale Stano, Alfonsina Ramundo-Orlando

**Affiliations:** 1 Institute of Translational Pharmacology-CNR, Rome, Italy; 2 Department of Information Engineering, Electronics and Telecommunications, Sapienza University of Rome, Rome, Italy; 3 Institute of Microelectronics and Microsystems-CNR, Rome, Italy; 4 Department of Med. Radiation Eng. Science and Research Branch, Islamic Azad University, Tehran, Iran; 5 Department of Biology and Environmental Science and Technology (DiSTeBA), University of Salento, Lecce, Italy

## Abstract

Among the many biological effects caused by low intensity extremely high frequency electromagnetic fields (EHF-EMF) reported in the literature, those on the nervous system are a promising area for further research. The mechanisms by which these fields alter neural activity are still unclear and thus far there appears to be no frequency dependence regarding neuronal responses. Therefore, proper *in vitro* models for preliminary screening studies of the interaction between neural cells with EMF are needed. We designed an artificial axon model consisting of a series of parallel RC networks. Each RC network contained an aqueous solution of lipid vesicles with a gradient of potassium (K^+^) concentration as the functional element. We investigated the effects of EHF-EMF (53.37 GHz–39 mW) on the propagation of the electric impulse. We report that exposure to the EHF-EMF increases the amplitude of electrical signal by inducing a potassium efflux from lipid vesicles. Further, exposure to the EHF-EMF potentiates the action of valinomycin – a K^+^ carrier – increasing the extent of K^+^ transport across the lipid membrane. We conclude that exposure to the EHF-EMF facilitates the electrical signal propagation by increasing transmembrane potassium efflux, and that the model presented is promising for future screening studies of different EMF frequency spectrum bands.

## Introduction

Over the past few decades, considerable evidence has shown that non-thermal exposure to low intensity extremely high frequency electromagnetic fields (EHF-EMF), in the range of 40 GHz-130 GHz^1^, can induce biological changes both *in vivo* and in *vitro*, including gene expression^2^, cell proliferation^3^, and nerve cell function^4^. It can also be conducive to the treatment of neurological disorders^5,6^. Specifically, changes in neuronal firing rate and plasma membrane properties were reported in mouse cortical slices^7^. One possible explanation for these effects may come from recent investigations into the prevalence and role of macromolecule-bound water, particularly inside and adjacent to the cell membrane. These point to strong specific absorption in this frequency spectrum band^8^. Therefore, the neurons in the central nervous system are likely to be the most sensitive candidates, as exposure to EHF-EMF may excite or suppress neuronal activities through changes in the permeability of the nerve cell's axonal membranes to specific ions^9^. Thus far it appears that there is no frequency dependency regarding neuronal responses^10^, hence the need for proper *in vitro* models for preliminary screening studies of the interaction between neural cells and a wide range of EMF with different frequency spectrum bands.

Despite the enormous complexity of the nervous system, there are some aspects of neuron function that can be understood from simple physical principles. One of those aspects is the propagation of electrical impulses along neurons. Since neurons send information to one another via the primary electrical signal generated by nerve cells, that is, through the so-called action potential, they can be treated as classical electrical circuits^11^. Although the biological significance of electrical signaling differs from one class of neuron to the other, the wave shape of the signal is always the same, arising from changes in the permeability of the nerve cell's axonal membranes to specific ions^9^. Thus, electrical signals travel through neurons via ions. During the propagation of an action potential along a nerve fibre only passive transport is involved. This is considered to be in response to a gradient of the electrochemical potential, while active processes, which involve the flow of ions against the electrochemical potential, merely recharge the energy sources^12^.

In the present study, and for the first time, we have directly measured the electrical signals traveling through an artificial axon model composed of a RC-network circuit containing aqueous solution of lipid vesicles with a gradient of potassium (K^+^) concentration as the functional element. We investigated the real-time effects of EHF-EMF on the propagation of the electric impulse at a frequency of 53.37 GHz – a frequency which is used for therapeutic treatment of over fifty medical conditions^13^.

We provide direct evidence that EHF-EMF facilitates the electrical signal propagation by increasing transmembrane K^+^ efflux, and that the artificial axon model presented is promising for future screening studies to test the interactions of different EMF frequencies.

## Results

### Signal character of the axon model was similar to that of neural one

**Figure 1:**
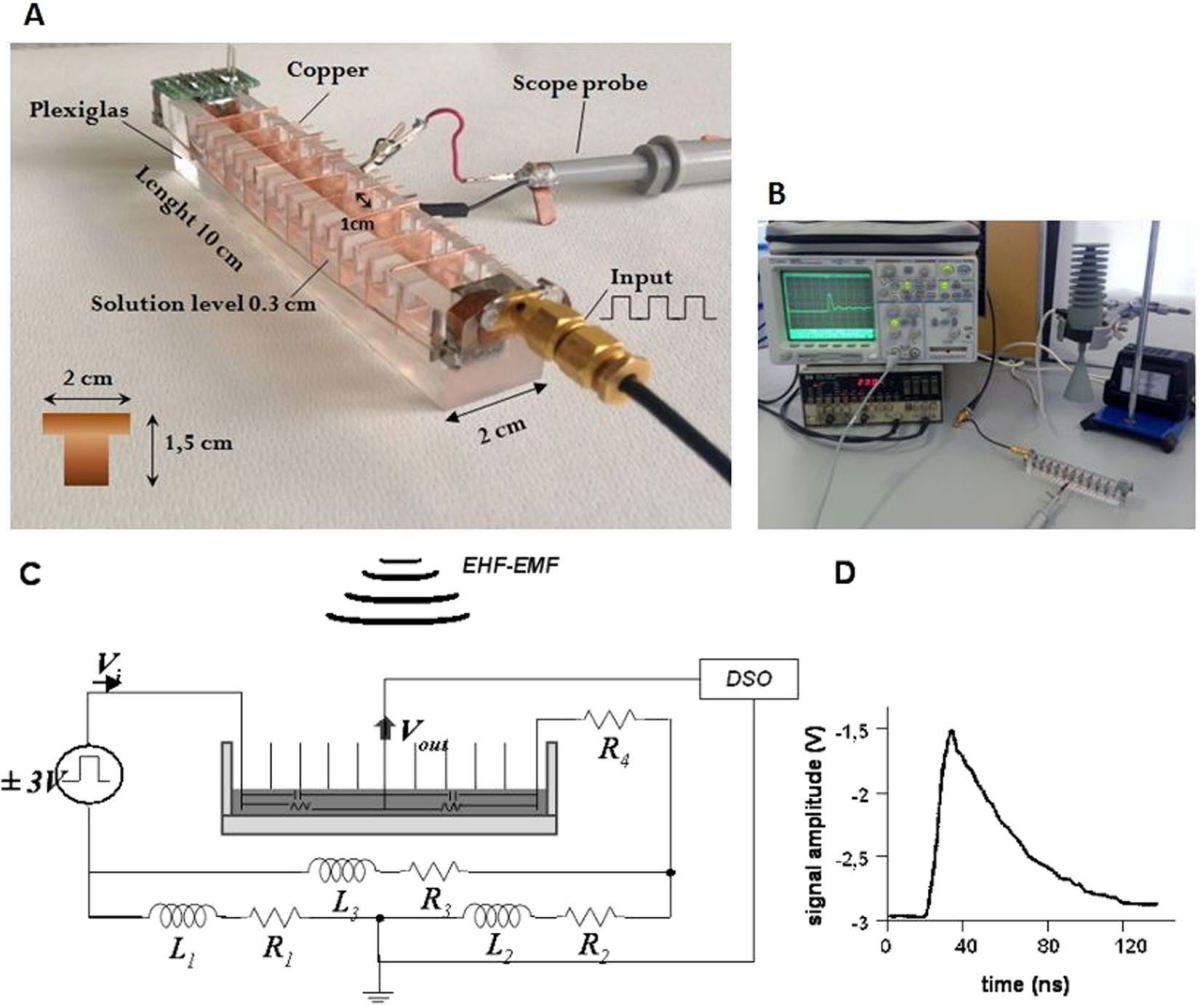
Overall experimental set up. (**A**) Image of the artificial axon model and the connections. (**B**) Electrical apparatuses with axon model and 53.37 GHz source (conical horn antenna). (**C**) Block diagram illustrating the equivalent electric circuit, where R_1_ = R_2_ = 0.0035 Ohm, R_3_ = 0.007 Ohm, R_4_ = 650 Ohm; and L_1_ = L_2_ = 60 nH, L_3_ = 120 nH. The resistance and capacitance of the aqueous solution is indicated. Symbol of the antenna (EHF-EMF) is also shown. (**D**) An example of electrical signal measured in the axon model containing 3 mL of aqueous solution (5 μM glycine-20 mM sucrose, pH 7.0 ± 0.5) containing 20 mM K_2_SO_4_. Oscilloscope settings: X-axis 100 ns/div, Y-axis 200 mV/div, averaging over 16 acquisitions and time resolution 2.5 ns. Post acquisition normalizing of the raw trace was applied.

**Figure 2:**
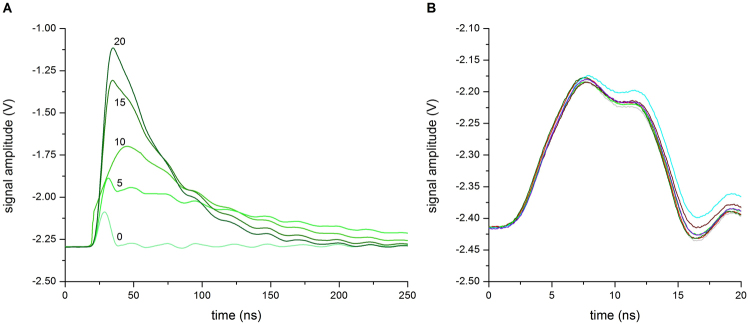
Changes of electrical signals dependent on K_2_SO_4_ concentrations. (**A**) Typical traces recorded in 3 mL of aqueous solution (5 mM glycine-200 mM sucrose, pH 7.0 ± 0.5) containing large increases in K_2_SO_4_ concentrations (mM). Oscilloscope settings: X-axis 100 ns/div, Y-axis 200 mV/div, averaging over 16 acquisitions and time resolution 0.025 ns. (**B**) Typical traces recorded in 3 mL aqueous solution (5 mM glycine-200 mM sucrose, pH 7.0 ± 0.5) containing low increases in K_2_SO_4_ concentrations. The colored lines are traces obtained with the following concentrations (mM): 0 (grey), 0.025 (red), 0.04 (olive), 0.1 (green), 0.2 (violet), 0.25 (wine) and 0.5 (cyan). Oscilloscope settings: X-axis 5 ns/div, Y-axis 50 mV/div, averaging over 16 acquisitions and time resolution 0.025 ns. Post acquisition normalizing of the raw traces was applied in all cases.

In order to check the reliability of our axon model (Fig. 1A) the electrical signal measurements were made of an aqueous solution (5 µM glycine-200 mM sucrose, pH 7.0 ± 0.5) containing different K_2_SO_4_ concentrations from 0 mM to 20 mM (Fig. 2A). Conductivity values were estimated to range from 0.33 μS/cm to 6140 μS/cm, respectively. Considering transmission speed and pulse shape parameters (peak pulse, rise-time, fall-time) these tests resulted in a signal shape similar to action potential commonly recorded from neuron tests, particularly at 20 mM and 15 mM K_2_SO_4_ concentrations with the amplitude around 1 V. As expected, decreasing K_2_SO_4_ concentrations from 10 mM to 0 mM resulted in the signal amplitude decreasing accordingly, reaching the lowest amplitude (approximately 0.2 V) at 0 mM (Fig. 2A).

### EHF-EMF induces an efflux of potassium from lipid vesicles

To evaluate how EHF-EMF could influence the signal transmission along the axon model, vesicle encapsulating potassium ions were added to the axon model solution. Vesicles prepared by the extrusion method were unilamellar^14^, thus insuring that only one bilayer is formed and having a similar structure to the cell membrane separating the inner water phase from the outer. The gel-filtration chromatography allowed the complete removal of non-encapsulated K^+^, so that a potassium gradient was established across the vesicle membrane.

The results of the ICP-MS analysis showed that vesicles prepared with aqueous solution (5 μM glycine-20 mM sucrose pH 7.0 ± 0.5) in the presence of K_2_SO_4_ concentration of 20 mM, 40 mM and 60 mM resulted in accord with an increasing encapsulation rate (internal K^+^ concentration of 0.31 mM, 0.36 mM and 0.48 mM, respectively). Throughout this work we report data obtained only from vesicles prepared with a starting solution of 60 mM K_2_SO_4_ since they have a higher K^+^ encapsulation rate than those prepared with starting solutions at lower K_2_SO_4_ concentrations (20 mM and 40 mM). This means that if a complete dissociation of K_2_SO_4_ in 2 K^+^ and SO_4_^2−^ occurs inside the vesicles^15^, the concentration range of K^+^ released into the axon model external solution should be from 0 mM to 0.48 mM, corresponding to K_2_SO_4_ concentrations from 0 mM to 0.24 mM.

To verify the reliability of the axon model at these low K_2_SO_4_ concentrations, electrical signals were collected for aqueous solutions (5 µM glycine-200 mM sucrose, pH 7.0 ± 0.5) containing K_2_SO_4_ concentration from 0 mM to 0.5 mM. Typical electrical signals recorded in these conditions are shown in Fig. 2B. A broadening of the shape was observed.

The signal was also characterized by over and under shoots with low amplitude (about 0.3 V) and the presence of two consecutive peaks. This was due to the attenuation effect on the signal transmission caused by the presence of obstructions (i.e., the copper plates) in the local circuit. Since they were separated by very short time (i.e., less than 4 ns), these peaks belong to the same signal, a condition reported in literature when classifying, for instance, two spikes belonging to the same discharge^16^. However, this peculiarity in the shape did not affect the intrinsic characteristic of the electrical signal itself, as confirmed by analysis using an oscilloscope with spectral analysis option (RTO2044, Rohde & Schwarz). For solutions with increasing K_2_SO_4_ concentrations from 0.1 mM to 10 mM the same frequency content was present, with increasing amplitude related to increasing potassium concentrations (see Supplementary Fig. S1).

Since lipid membranes are good insulators – conductance per unit area is approximately *g* = 10^−13^ Ω^−1^/m^2^ – we firstly checked if empty vesicles themselves, those not containing K_2_SO_4_, could influence the electrical signal. Experiments were conducted on three different empty vesicle preparations and electrical signal records were collected during both sham and exposed conditions. In all cases no difference in the parameters of the recorded signal was observed (data not shown).

The same experiments and analysis were performed on the axon model when containing solution of vesicles encapsulating K^+^, in which a potassium gradient was established across the vesicle membrane. Vesicles were added to the axon model and signal recorded during 30 min in sham condition (i.e., when the field was turned off). Again no difference was observed in the electrical signals in any of the cases (see Supplementary Fig. S2). These were characterized by low amplitude, less than 0.3 V, similar to those tested with the external aqueous solution (5 µM glycine-200 mM sucrose, pH 7.0 ± 0.5) at 0 mM K_2_SO_4_; thus indicating a good stability of vesicle preparations with no simple diffusion of potassium ions from the interior of vesicles occurring, a prerequisite for successful investigation of the effects induced by EHF-EMF.

**Figure 3:**
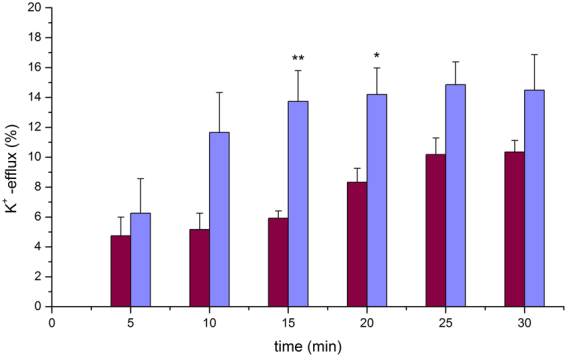
EHF-EMF and valinomycin effects on efflux of potassium from vesicles. Plot of normalized data as the mean ± SD . of six exposed (wine-colored bars) and four valinomycin added (violet-colored bars) samples is shown. The percentage rate of potassium efflux was determined by the ratio of K_2_SO_4_ concentrations on the sequence of traces recorded during the experiment and after the addition of Triton-X100 (i.e., taken as 100%) at the end of experiment. At time 15 and at time 20 a *t-*test two tailed was performed between the means ± SD. of the EHF-EMF exposed samples versus the valinomycin samples, the resulting *p* for each is indicated in the figure: **p = 0.034, and *p = 0.047, respectively. [K_2_SO_4_] = 0.28 mM ± 0.16 (n = 6 exposed) and [K_2_SO_4_] = 0.21 mM ± 0.12 (n = 4 valinomycin) calculated by calibration curve.

When the same experiments and analysis were performed on the axon model containing solution of vesicles encapsulating K^+^ subjected to 30 minutes of EHF-EMF exposure a slight difference was observed on the electrical signal records, which depended on the exposure time. In order to normalize the data obtained from six different vesicle preparations at the end of each exposure the electrical signal was recorded after the addition of detergent Triton-X100. The concentration of K_2_SO_4_ was calculated using the calibration curve (see Supplementary Fig. S3) and the data was normalized by calculating the concentration at 100% of K_2_SO_4_ released from the interior of vesicles after the addition of the detergent. The percentage change of K_2_SO_4_ concentration – considered thereinafter as the rate of K^+^ efflux – increased progressively from 0 to 10.2 ± 2.2% in 25 minutes of exposure (Fig. 3 wine-colored bars); no further significant increase of the efflux rate resulted even after prolonging the experiment by 5 minutes.

### Effect of valinomycin on the efflux of potassium

To functionalize the vesicles we created a transmembrane potential by adding valinomycin to the solution in the axon model. This is a 1.1 kDa peptide, which is capable of forming K^+^-selective pores^17^ through which K^+^ is transferred across the cell membrane. In our vesicles, the passage of potassium ions through the valinomycin pores, guided by the concentration gradient, allowed the establishment of a transmembrane potential^18^. Thus, the same experiments and analysis were performed on the axon model containing vesicles encapsulating K^+^ with valinomycin immediately added and signals recorded for 30 minutes. Also in this case, to normalize the data obtained from four different vesicle preparations, at the end of each exposure the electrical signal was recorded after the addition of detergent Triton-X100. Valinomycin appeared to have stimulated transmembrane potassium transport; the rate of K^+^ efflux rose from 0 to 13.7 ± 4.0% in 15 minutes (Fig. 3 violet-colored bars). Similar activation time is reported in literature^19^ for the approximate molar ratio of valinomycin to lipid of 1.8 × 10^−3^ in the same order used in this work. However, no further significant increase of the rate of K^+^ efflux resulted even after prolonging the experiment time by 15 minutes (Fig. 3 violet-colored bars). This indicates that a steady state exchange was established between the internal K^+^ and external H^+^, as reaffirmed in literature^15^. In fact, since it is a counter-anion system, it is unlikely that the doubly charged SO_4_^2−^ would come out because it normally moves only very slowly through the phospholipids bilayer and, importantly, it is not significantly affected by the addition of valinomycin^15^.

### EHF-EMF facilitates transmembrane potassium efflux

**Figure 4:**
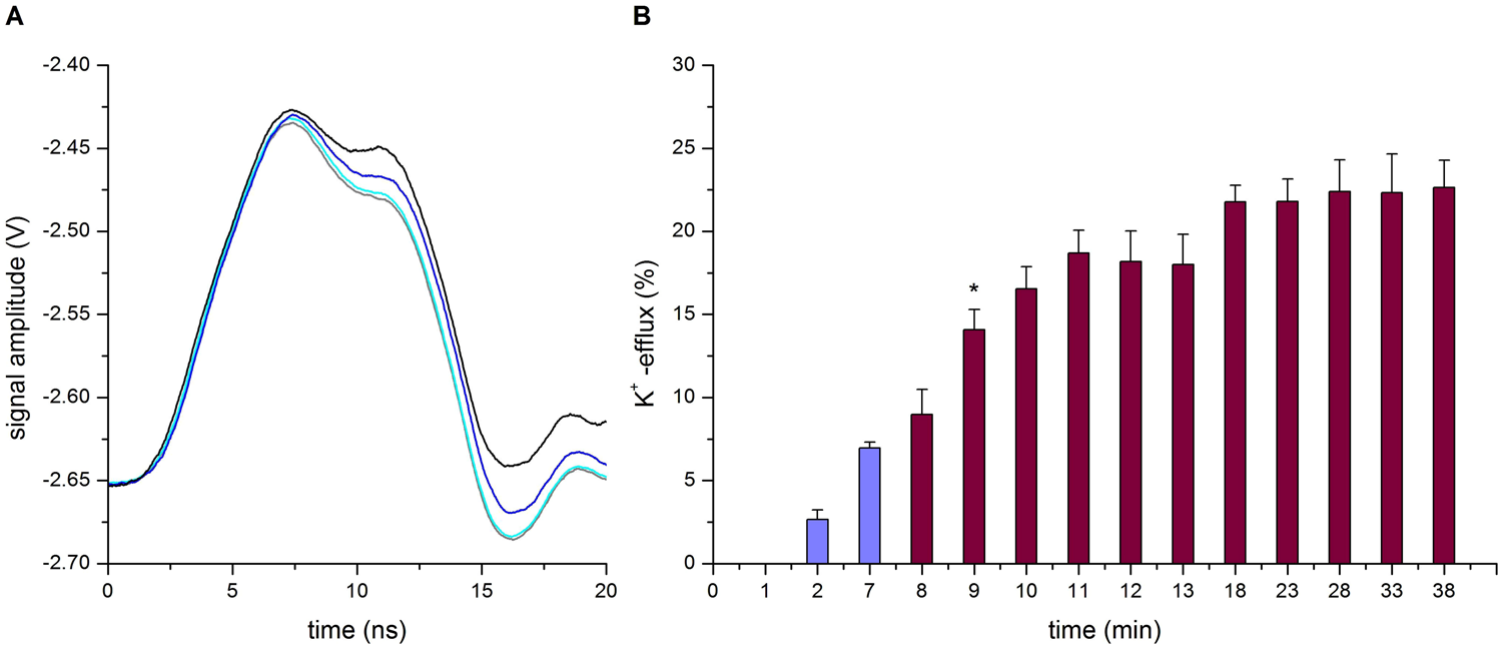
EHF-EMF facilitates transmembrane potassium efflux. (**A**) An example of the sequence of electrical signals recorded during EHF-EMF exposure in the presence of valinomycin added. The colored lines are traces obtained with the following treatment: VAL added at time 0 (grey), at time 7 min (cyan), at time 28 min (blue) and after addition of Triton-X100 at the end of experiment (black). Oscilloscope set and post acquisition as in Fig. 3B. (B) Plot of normalized data as the mean ± SD. n = 6 of vesicle preparations is shown. The percentage rate of potassium efflux was determined by the ratio of K_2_SO_4_ concentrations on the sequence of electrical signals recorded with valinomycin added EHF-EMF exposed. The EHF-EMF exposure started immediately after records of valinomycin at 7 min (violet-colored bars); total field exposure time was 30 min (wine-colored bars). A significant difference between the means ± SD. at time 8 and that at time 9 is indicated by the t-test two tailed *p = 0.028. [K_2_SO_4_] = 0.21 mM ± 0.06 (n = 6) calculated by calibration curve.

To further explore how EHF-EMF affects the signal transmission along the axon model, we examined vesicles encapsulating K^+^ with a transmembrane potential created by added valinomycin. In this case each experiment lasted 38 min; valinomycin was added at the start and incubated for 7 minutes before EHF-EMF exposure. An example of the changes in the electrical signals recorded during the experiment is shown in Fig. 4A. Again in order to normalize data obtained from six different vesicle preparations at the end of each exposure the electrical signals were recorded after the addition of the detergent Triton-X100. Interestingly, valinomycin appeared to be more active in stimulating transmembrane potassium transport in the presence of the field than alone; K^+^ -efflux was 14.8% ± 3.0% (t = 25′) when alone (Fig. 3 violet-colored bars) and it increased significantly (p = 0.009) to 21.8% ± 2.7% (t = 23′) in the presence of EHF-EMF (Fig. 4B violet-colored bars). The K^+^ -efflux was 22.6% ± 3.2% (t = 38′) after an additional exposure of 15 minutes without any significant increase (p = 0.610 for t = 23′ as compared to t = 38′); thus indicating that also in this case a steady state had been achieved (Fig. 4B violet-colored bars).

Finally, it should be noted that although the pool of measured samples (n = 6) allowed a meaningful comparison between different experimental conditions (EHF-EMF only, valinomycin only, and valinomycin in the presence of EHF-EMF), a large set of measurements is needed to corroborate these observations^20^.

### EHF-EMF energy absorbed inside the axon model

**Figure 5:**
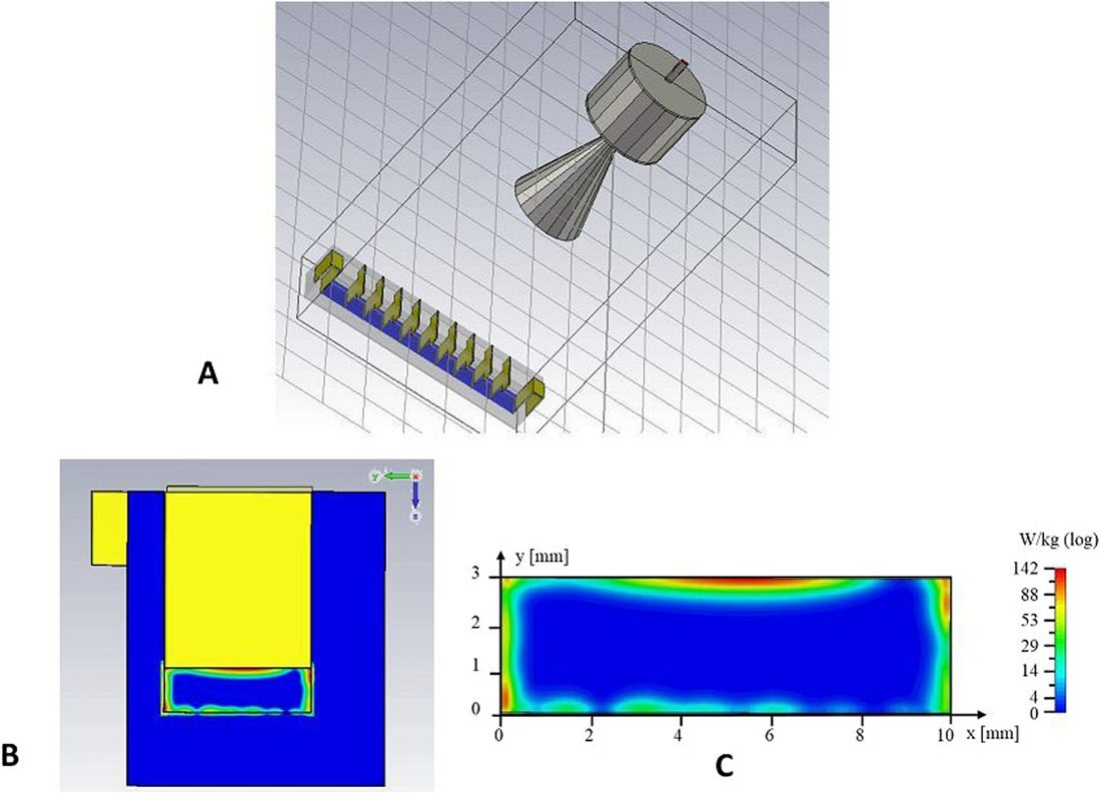
Numerical simulation. (**A**) Model of exposure set up including the conical horn antenna and the axon model. (**B**) View of the well in the centre of axon model: the aqueous solution (blue) and the copper plates (yellow). (**C**) Magnification of aqueous solution in (B) with absorbed power (SAR) distribution is indicated in W/Kg, log scale.

To achieve a quantitative understanding of the transmembrane potassium transport stimulation in the presence of EHF-EMF, the specific absorption rate (SAR) was quantified and correlated with the observed phenomenon. Numerical simulation data (Fig. 5) demonstrated that, the penetration depth of the electric field, which is linked to the high attenuation of the field in the water, is about 0.33 mm at 53.37 GHz. Higher SAR values resulted at the top, bottom and sides of the wells. However, a very uniform distribution of SAR was present in the central area of the aqueous solution (Fig. 5C). The local SAR value of 1.1 W/kg (see Supplementary Fig. S4) was calculated at the vertical centre of the aqueous solution, where vesicles are most likely dispersed over the entire time of the experiment; this SAR value is within the recommended SAR threshold value (i.e., 1.6 W/kg) below which there is no significant temperature elevation that would cause biological effects^21^.

**Figure 6:**
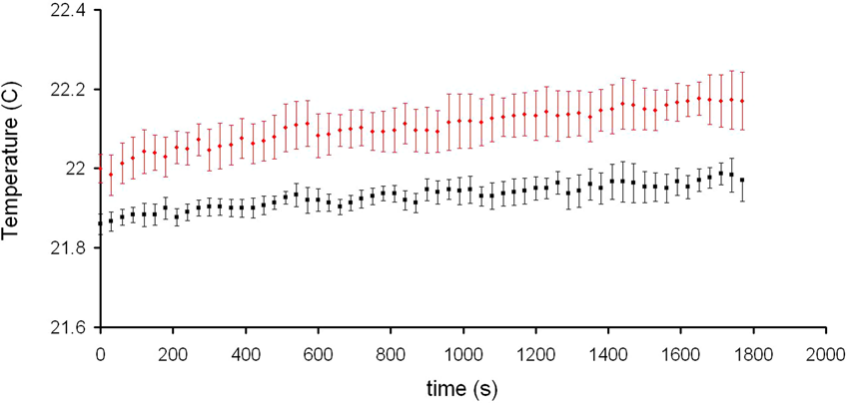
Temperature dynamics recorded in 3 mL of the aqueous solution (5 µM glycine-200 mM sucrose, pH 7.0 ± 0.5). The probe tip was placed in the well located at the centre of the axon model away from bottom and sides of Plexiglas channel (Fig. 1A) and away from the surface of the solution. Data are the mean ± SD. n = 3 in sham (black) and exposure (red) conditions.

The expected negligible elevation of temperature from this SAR level was confirmed by direct temperature measurements. The temperature dynamics recorded during three sham and exposure experiments is shown in Fig. 6. The temperature difference between the sham and exposed samples at the end of experiments was +0.08 C. This was well below the value that literature considers to be the threshold of what should be considered thermal^22,23^.

## Discussion

This study shows that exposure to the EHF-EMF facilitates the electrical signal propagation in a lipid vesicle construct by increasing transmembrane potassium efflux in an artificial model of the neuron axon. To our knowledge, this is the first time that an electrical biomimetic circuit is has been combined with an artificial membrane model in order to obtain insights into the effects of EMF. According to the transmission model, a nerve can be represented as a lumped element transmission line of RC elements^12^. In our axon model several compartments resulted in series when copper plates were inserted along it; this spacing, together with the area of these plates, creates a series of capacitors in the pF range (Fig. 1C). Taking the level of the aqueous solution, (3 mm) in the axon model, and the electric parameters of this circuit into account we calculated, as indicated in^11^, a conductivity value of 0.03 S/m. This was similar to the value as reported for ovine axon in literature^24^. This means that in our model a capacitance value approximating that of a neural axon can be reproduced.

With accurate potassium concentration measurements we provide direct evidence that exposure to EHF-EMF affects the ions permeability across the lipid membrane (Fig. 3 wine-colored bars). Although thermodynamics concerns systems in equilibrium and propagating pulses are dynamical phenomena, it is hypothesized that thermodynamic theory could explain how pulses propagate in nerve membranes^25^ where a fundamental role has been given to membrane lipids, specifically to transient geometric changes (i.e., volume, area, and thickness) of the membrane. This implies that the state of the membrane can be influenced not only by temperature but also by hydrostatic pressure and lateral pressure in the membrane plane^26^. Further, in an attempt to explain how some form of ion flow is involved in nerve signal propagation, an increase of several orders of magnitude in the membrane ion permeability in the vicinity of its fluid-gel transition has been suggested^25,27^. In this context, it has been found that the lateral pressure dynamics of the membrane is significantly influenced by EHF-EMF^28^, and that this membrane property is highly sensitive to even small changes in membrane composition. Further, direct effects of EHF-EMF on the voltage-sensitive channels in the neuronal plasma membrane^7^ and an increase in permeability due to rearrangement of membrane phospholipids structure have been indicated^29^.

Choi *et al*.^30^ suggested that the best neuronal mimic would be an excitable vesicle composed of ion channels, an ion pump and gap junctions^31^. Here we present an alternative approach based on lipid vesicles and generation of a transmembrane potential in an attempt to examine how direct effects on bilayer permeability resulted in changes of the electrical signal due to the modulation of potassium ions efflux. This was accomplished by adding the ionophore valinomycin during the experiments (Fig. 3 violet–colored bars). This dodecadepsipeptide functions as a potassium-specific transporter and facilitates the movement of K^+^ through the lipid membranes by forming K^+^ -selective pores in the vesicles.

We demonstrated that exposure to EHF-EMF potentiates the extent of transmembrane potassium transport facilitated by valinomycin. In particular, K^+^ efflux increased about 10% (depending on the exposure time) (Fig. 3 wine-colored bars). EHF-EMF increases the K^+^ permeability across the lipid bilayer when valinomycin is absent and when it is present (Fig. 3 wine-colored bars *vs*. Figure 4B violet-colored bars). In the latter case, the K^+^ release occurs effectively despite a partial dissipation of the concentration gradient and despite the presence of a transmembrane potential (negative inside). Therefore, we conclude that EHF-EMF is very effective in altering the membrane structure (increasing K^+^ permeability), although a description of its action at the molecular or supramolecular levels remains elusive. In particular, potentially synergistic effects related to membrane organization and pore-forming peptide/proteins should be carefully looked into in the future.

Recently, ‘action potential’ shape analysis has been used as a valuable tool to classify the effect of toxins based on their mechanism of action^32^. Indeed the shape of an action potential can carry information concerning the state of the involved ion channels in the cell membrane and their relationship to cellular processes. For instance, the toxin quinine – a K^+^ channel blocker – induced a broadened shape effect on the action potential in rat glioma cells by reducing conductance of both K^+^ and Na^+^ channels^32^. Thus observed changes in signal shape could be associated with a change of ionic conductance in the axon model, which could be taken into account in the determination of the potassium efflux from the vesicles. On the other hand, evidence indicating that the shape of the ‘action potentials’ is determined by the status of ions channel in the surrounding membrane environment has been recently reported^25,32^. In a similar way our observed change in the signal shape could be determined by the action of field on the orientation of charged and dipolar molecules at the bilayer/water interface region of the bilayer^33^, thus determining the rearrangement of phospholipids in the bilayer, which results in an increase in the permeability of potassium ions across the vesicles.

Although sample heating is the most widely accepted mechanism of high frequency EMF interaction in biological systems, in our case we can safely hypothesize that the observed effects were mediated through non-thermal mechanisms (Figs 5 and 6). Recently, similar mechanisms were proposed as an explanation of the effects of low intensity EHF-EMF on nervous tissue involving a direct interaction with the neuronal plasma membrane^7^. Further non-thermal mechanisms were also suggested to explain the transient response of high frequency EMF on the electrical activity of the sural nerve *in vivo*, which appears to be specific to the field because the radiant heating did not reproduce this effect^34^. Thus our results suggest subtle specific effects, which do not depend on the thermal energy imparted by the EHF-EMF on the axon model.

## Conclusions

We have developed an artificial axon model in which we introduced lipid vesicle solutions loaded with K_2_SO_4_, with or without valinomycin pores in the lipid bilayer. When exposing this set-up to a continuous wave 53.37 GHz for up to 30 min, the K^+^ ions inside the vesicles pass through the lipid membrane into the surrounding medium. Furthermore, the EHF-EMF exposure enhances the leakage that occurs through the valinomycin pores. Our physical model, created by a ten parallel RC in which the resistance is due to the inner solution and the capacitance by the adjacent copper lamina, displays a similarity with the isopotential electrical circuit of a real axon, but mimics only a narrow slice of the complex behavior of the real axon.

This scaled-down model of a nerve cell axon can be modified in many ways in order to investigate a number of questions related to both membrane physiology, and possibly provide new insights. As a means it can be compared to that of using fully differentiated central nervous system neurons, which, as a mixture of cell types, makes neuronal-based electrical recordings somewhat dubious. It also could help in the investigation of how external agents, including various forms of EMF, influence the nerve cell membrane and thus indicate possible effects on a nervous system.

As any model, however, the artificial axon model here presented has functional limitations that should be kept in mind. In particular the mechanism of action potential generation clearly differs from the biological one. Nonetheless, the possibility of modular modifications (change the lipid composition, adding membrane proteins, introducing biochemical elements inside the vesicles or in the external medium) offers interesting opportunities for future studies.

Our innovative hybrid biomimetic system could be valuable in preliminary studies, e.g. those when methodology or mechanistic studies need to be established to optimize *in vitro* and *in vivo* studies on neurons (in particular) or any other type of biological cell (in general).

## Materials and Methods

### Chemicals

All chemicals used in this work were used without any pre-treatment. L-a-phosphatidylcholine from egg yolk (Egg-PC, Type XVI-E, >99%), potassium sulfate (K2SO4, >99%), valinomycin (VAL > 98%) and Triton X-100 (TM-X-100, >99%) were purchased by Sigma-Aldrich (USA). Glycine (Gly, 50049, >99%) and sucrose (84099, >99.5%) were purchased by Fluka Chemie GmBH (Switzerland).

### Artificial axon model

The artificial axon apparatus (Fig. 1A), made in house, is a rectangular Plexiglas channel with inside dimensions 10 cm long × 1 cm wide × 2 cm deep. The elongated geometry is similar to a nerve string. The electrical stimulus is launched from a copper plate at one end of the channel and terminated at a copper plate at the opposite end. The ground return between stimulus (signal generator) and termination consists of two heavy gauge wires, one running down each side of the Plexiglas. A series of grooves spaced 1 cm apart along the top of the long sides of the channel support nine vertical copper plates (1.5 × 2 cm × 0.5 mm) used to form ten separate wells; the plates are not grounded. The electrical signal was measured at the fifth intermediate plate. During testing the channel would be filled with an appropriate aqueous medium.

The electrical signal, connected to the first plate of the axon model (Fig. 1A), was a rectangular pulse train generated by an HP 8112 A Pulse Generator 50 MHz (Fig. 1B). The signal period was 2 µs, with signal levels –3 V for 99.5% of the period and +3 V for 0.5% of the period (10 nsec). The last plate of the axon model is connected to a 650 Ω resistor, the other terminal of which is connected to the ground return wires. The calculated resistance from the first to last plate was of about 92 kΩ.

The output was recorded by connecting an oscilloscope (Agilent 54642D bandwidth 500 MHz and maximum sampling rate of 2GSa/s) to the fifth vertical copper plate from the input (Fig. 1A) and to one of the ground wires via an Agilent 10073 C probe; this position was considered to be the optimum position regarding direct exposure to radiation and isolated from any noise arising from the pulse generator connection. Oscilloscope settings (indicated in the figures legends) were optimized to observe the best resolution of the signal waveform for observing small variations in amplitude and/or width of the signal.

The equivalent electrical circuit of the experimental situation is depicted in Fig. 1C. Resistors (R1, R2, R3, R4) and inductors (L1, L2, L3) are part of the experimental circuit, not the Axon model. The presence of ionic solution between each two plates gives rise to a resistance and capacitance, both variables due to the presence of potassium ions. The typical signal from the axon model with an aqueous solution (5 µM glycine-20 mM sucrose, pH 7.0 ± 0.5) containing 20 mM K_2_SO_4_ is shown in Fig. 1D.

### EHF-EMF exposure system

The axon model was exposed to EHF-EMF at 53.37 GHz using a generator with conical horn antenna (IMG-53.37, Micro Med Tech, Russia) with a maximum diameter of 34 mm and an output power of 39 mW. The antenna (Fig. 1B) was centered directly above the axon model (Fig. 1A) at a distance of 8 cm from the top of Plexiglas channel. This distance satisfies generally accepted conditions for operating in the far field condition^35^. Thus the axon model was in the irradiative region of the antenna and a uniform distribution of electromagnetic field strength incident on the axon model could be assumed.

This assumption was also supported by the numerical simulation analysis performed by using CST Microwave Studio software (CST-MWS2008, GmbH, Darmstadt-Germany) based on Finite-Integration-Technique (FIT)^36^. Analysis showed that the radiated electric field was not significantly altered by the presence of the axon model. This software solves the electromagnetic problem by dividing the domain under study into elementary cells (0.5 × 0.5 × 0.5 mm) a dimension much smaller than the minimum wavelength (λ_0_/10). The geometries of the circular horn antenna and the axon model including the internal aqueous media were carefully reproduced (Fig. 5A) and simulations were carried out considering the electric parameters of the materials at the working frequency (53.37 GHz) reported elsewhere^36,37^.

The absorbed power distribution inside aqueous media contained in the axon model (Fig. 5B,C) was calculated as local specific absorption rate (SAR), expressed in W/kg, by using the values of electric field (E) evaluated in the media and applying the formula (1):

1 $$SAR=\frac{1}{2}\frac{\sigma }{\rho }{|E|}^{2}$$

where σ is the effective conductivity and ρ is the density of the medium. The complex permittivity of the aqueous medium at 53.37 GHz is *ε*_*c*_ = 11.76 − j24^38^, which corresponds to a conductivity σ of 71.16S/m, *ρ* is 1000 kg/m^3^ and the wavelength (λ) in the medium is about 1.39 mm. The SAR, numerically calculated in the well solution located at the centre of the axon model, is shown in Fig. 5C.

Accurate temperature measurements inside the samples in the axon model were taken using a flexible implantable thermocouple probe (Physitemp Instruments, Inc. Type IT-18) of 0.06 cm diameter, having a 0.1 s time constant and resolution of 0.01 °C. The probe tip was placed in the well located at the centre of the axon model away from bottom and sides of Plexiglas channel (Fig. 1A) and away from the surface of the solution.

### Artificial axon exposure protocol

The exposure protocol was as follow: the axon model was partially filled, to a depth of 3 mm, with aqueous solution of 5 μM glycine-200 mM sucrose (pH 7.0 ± 0.5) at 3 mL final volume and after the insertion of copper plates, leveled using a circular spirit level, and immediately connected to the signal generator. The oscilloscope recordings were made both when 53.37 GHz radiation was present (i.e., exposed sample) and when it was absent (i.e., sham sample). A typical duration of the experiment was 30 minutes, as generally reported for *in vitro* studies of exposure to EHF-EMF^10^.

### Lipid Vesicle preparation

Lipid vesicles were prepared by the extrusion method^14^. Briefly, a lipid film of L-a-phosphatidylcholine egg-PC (10 mg) was suspended in 1 mL of 5 μM glycine-20 mM sucrose (pH 7.0 ± 0.5) containing different concentrations of K_2_SO_4_ (20, 40 and 60 mM) to yield a lipid concentration of 13 mM. After ten freeze-thaw cycles in liquid nitrogen (thawing at 42 °C), vesicles were extruded (20 times) through polycarbonate membrane (Whatman) of pore size 400 nm, using a LiposoFast small-volume extruder (Avestin, Ottawa, Canada). The average size of resultant vesicles was 430 ± 20 nm, and no formation of aggregates was detected by measurements with dynamic light scattering analyzer (LB500 Horiba, Ltd. Japan). All steps of the vesicle preparations and exposure were performed at ambient room temperature, which is well above the gel-liquid phase transition temperature of egg-PC (Tc = −7 °C), in order that the phospholipids were in their fluid phase. Moreover, egg-PC vesicles are stable at the pH of our working solution as indicated elsewhere^39,40^.

### Transmembrane potential

In order to create a concentration gradient between the inside and outside of the vesicles, the non-encapsulated potassium was removed by filtration: lipid vesicles (1 mL) were loaded on a 20 cm Sephadex G-50 coarse (Pharmacia, Uppsala, Sweden) filtration column, pre-equilibrated with 5 μM glycine (pH 7.0 ± 0.5) containing an appropriate concentration of sucrose to make the osmotic pressure of the equilibration solution equal to that of the internal vesicle, thus avoiding vesicle disruption during separation. The vesicle-containing fraction was collected by measuring turbidity at 403 nm^41^, and immediately used for subsequent experiments.

In order to create a transmembrane potential, experiments with valinomycin added were done in the following steps. The vesicle-containing fraction (1.2 mL) was diluted in 3 mL of an equi-osmolar sucrose solution (5 μM glycine-200 mM sucrose, pH 7.0 ± 0.5) and poured into the axon model, then 5 µL of valinomycin (10 mg/ml, Sigma-Aldrich) was added to a final concentration of 15 mM. The approximate molar ratio of valinomycin to lipid was 2.9 × 10^−3^.

### Potassium efflux measurements

To measure the potassium efflux from the vesicles into the aqueous solution contained in the axon model we used the following protocol. On each oscilloscope trace (i.e., sample) the ratio between maximum and minimum amplitude values (a/b_sample_) was calculated (see Supplementary Fig. S5). In all cases a similar ratio (a/b_blank_) was calculated on a reference solution (i.e., blank) containing all components other than the agent under investigation. To estimate the unknown concentration of K_2_SO_4_ in the aqueous vesicle's samples a = (a/b_sample_ − a/b_blank_) was calculated and compared to a calibration curve measured with known concentrations of K_2_SO_4_ (see Supplementary Fig. S3). The percentage of K_2_SO_4_ leakage was determined by taking as 100% the K_2_SO_4_ concentration estimated in the vesicle samples after addition of Triton X-100 that breaks the vesicles and equilibrates the internal and external K_2_SO_4_ concentration.

To provide an experimental control of reliability of potassium encapsulation, overall quantities of K^+^, encapsulated inside the vesicle were measured, after vesicle lyses, by mass spectroscopy (ICP-MS) using an inductive plasma source (Agilent Technologies 7500c) with instrumental configuration Octupole Reaction System (ORS). In all cases the quantities of K^+^ measured by mass spectroscopy fitted that estimated as the K_2_SO_4_ concentration by the above mentioned calibration curve (reported as mean ± SD. in the figure legends).

## Electronic supplementary material

Supplementary Information
