# Supplementary material for: Extremely High Frequency Electromagnetic Fields Facilitate Electrical Signal Propagation by Increasing Transmembrane Potassium Efflux in an Artificial Axon Model
Source: Sci Rep. 2018 Jun 18;8:9299. doi: 10.1038/s41598-018-27630-8 (PMC6006430; doi:10.1038/s41598-018-27630-8)
Supplement: Supplementary file 1 — Supplementary Information [file 41598_2018_27630_MOESM1_ESM.pdf]

Extremely High Frequency Electromagnetic Fields Facilitate Electrical Signal Propagation by Increasing Transmembrane Potassium Efflux in an Artificial Axon Model

Simona D'Agostino, Chiara Della Monica, Eleonora Palizzi, Fabio Di Pietrantonio, Massimiliano Benetti, Domenico Cannatà, Marta Cavagnaro, Dariush Sardari, Pasquale Stano & Alfonsina Ramundo-Orlando

Supplementary Information

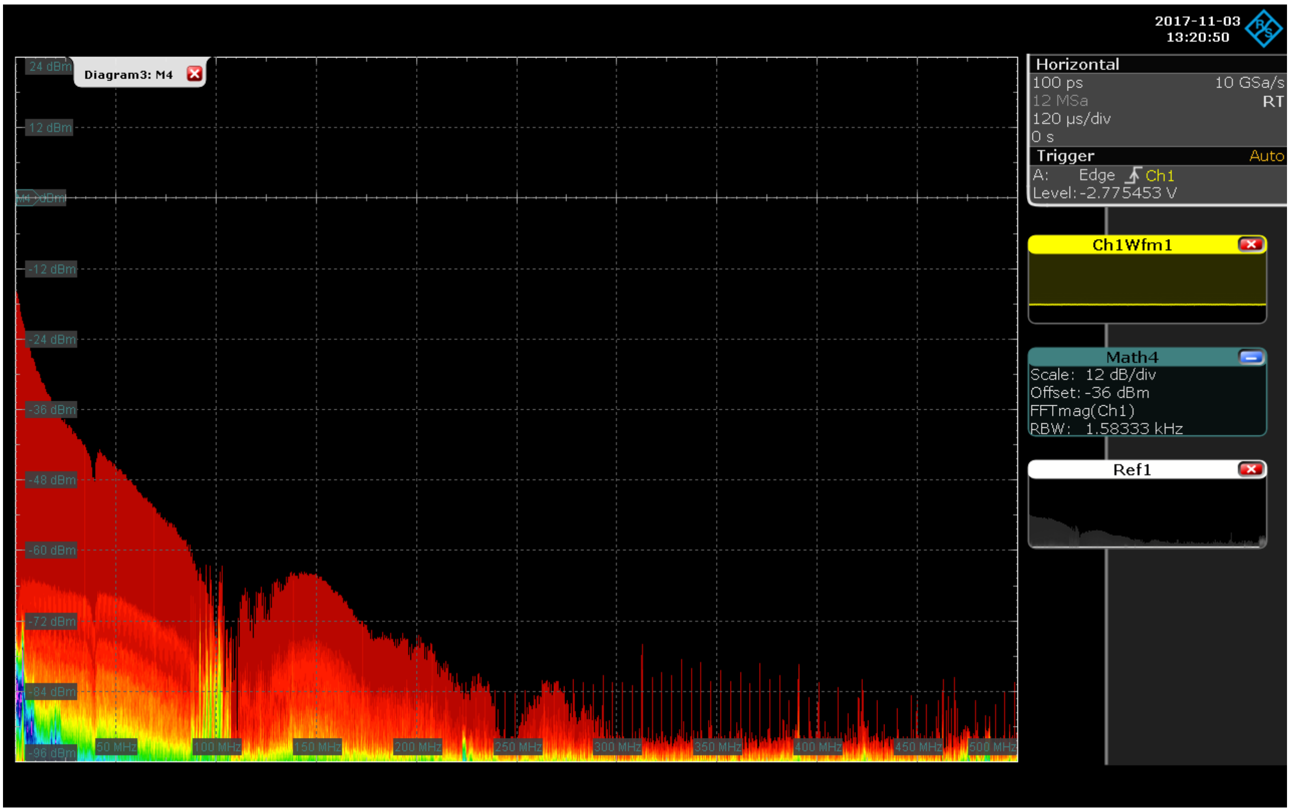

Figure S1. Spectral analysis of the electrical signal. Oscilloscope Fast Fourier Transform (FFT) measurements is shown. The image shows the frequency spectrum of the signal at the input plate (wine coloured) together with those resulting at each of the 10 vertical copper plates superimposed on it in differing colours. An aqueous solution of 5µM glycine-

200mM sucrose (pH 7.0 ± 0.5) containing 10mM K_2SO_4 was used. X-axis: Frequency. Y-axis: Amplitude.

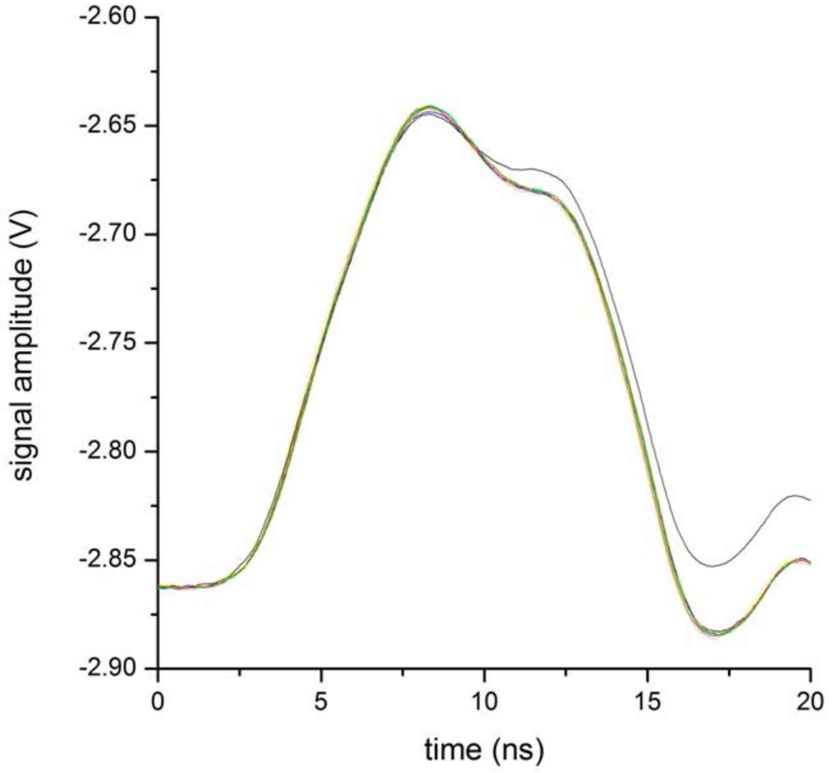

Figure S2. An example of the sequence of electrical signals recorded in the axon model containing vesicles preparation with [K_2SO_4] = 0.21 ± 0.06 (n=6) calculated by calibration curve under sham condition. The colored lines are traces obtained with the following treatment: at time 0 (Light Grey), at time 2 (Yellow), at time 7 (Cyan), at time 9 (Pink), at time 12 (Green), at time 13 (Olive), at time 18 (Orange), at time 28 (Blue) and after rupture with detergent TX-100 (black). Oscilloscope settings: X-axis 5 ns/div, Y-axis 50 mV/div, averaging over 16 acquisitions and time resolution 0.025 ns. Post acquisition normalizing of the raw traces was applied in all cases.

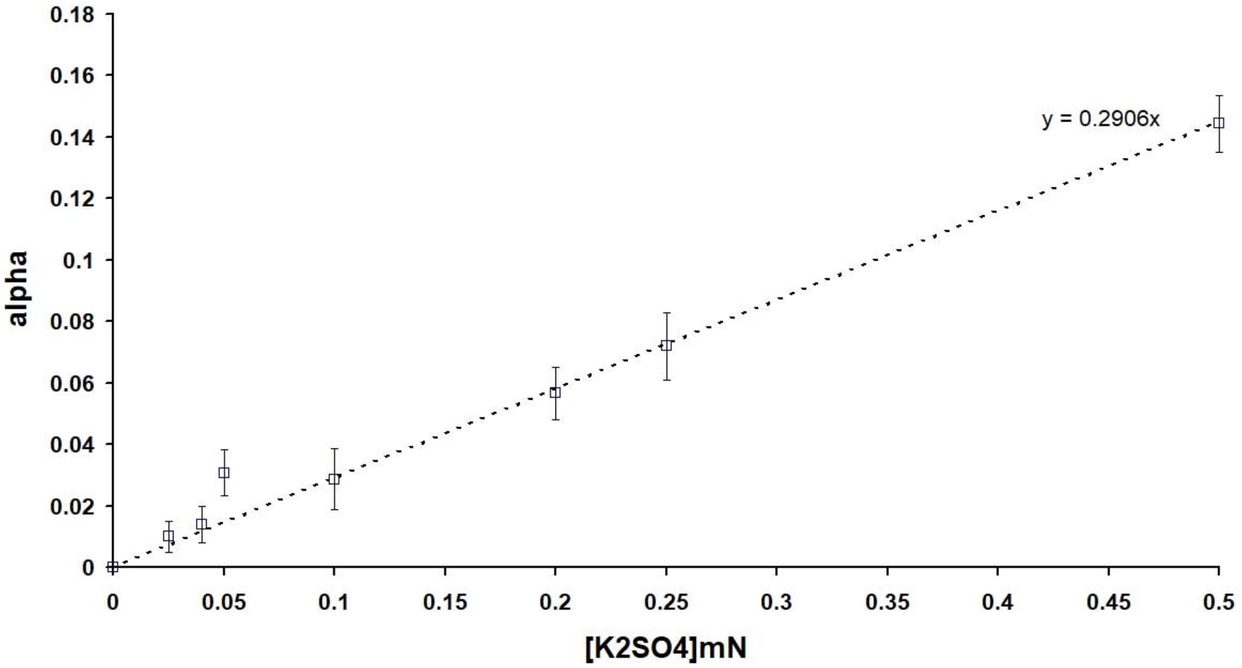

Figure S3. Plot of normalized data: alpha = ($(a/b)_{sample}$ - $(a/b)_{blank}$) (Y-axis) versus increasing concentrations of K_2SO_4 (X-axis) as displayed in Fig. 2B. Blank is aqueous solution of 5μM glycine-200mM sucrose without K_2SO_4. Data are the mean ± S.D. n=3 sets of measurements. The trend line and its formula are shown.

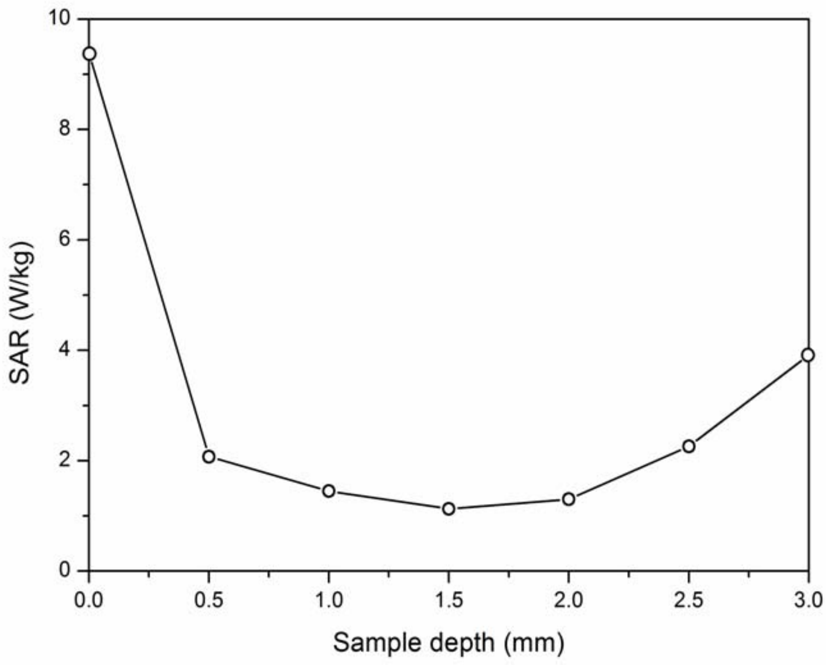

Figure S4. SAR distribution in the axon model. Local SAR value (W/kg) distribution averaged over a 0.5 mm strip as wide as the well surface (as in Fig. 5B) is reported as a function of the depth: from the figure it can be noted that the maximum SAR is 9.4 W/kg (at the surface) and the minimum value is 1.1 W/kg (in the centre of the well).

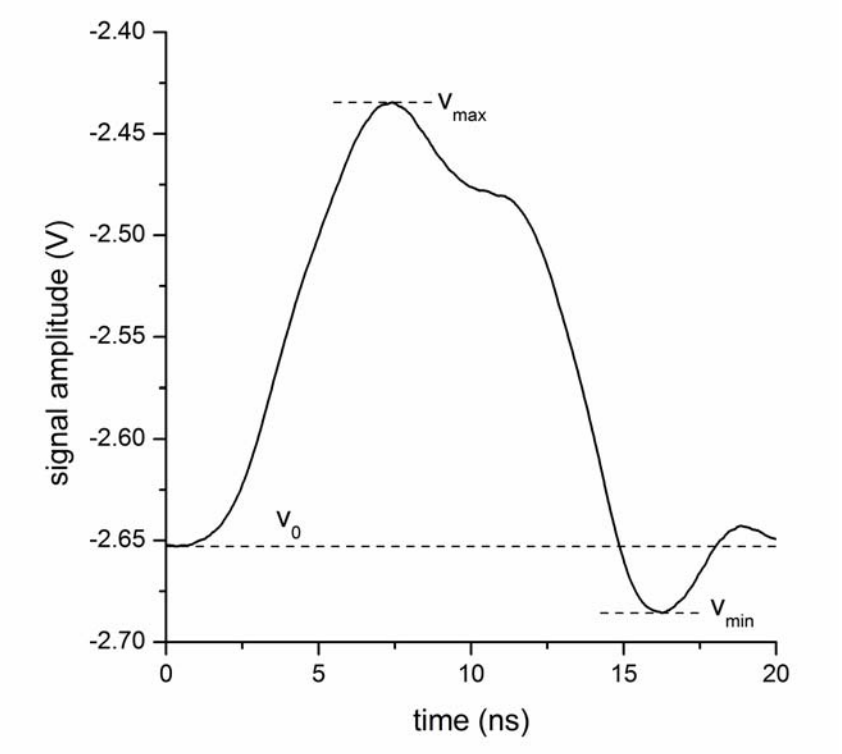

Figure S5. Calculation of the parameters *a* and *b* on a typical electrical signal, where $a = V_{max} - V_0$ and $b = V_{max} - V_{min}$. The trace is from the experiment as displayed in Figure 2B. Oscilloscope set and post acquisition as in Fig.2B.
